# Polycythemia-Related Proliferative Ischemic Retinopathy Managed with Smoking Cessation: A Case Report

**DOI:** 10.3390/ijerph19138072

**Published:** 2022-06-30

**Authors:** Shao-Yu Sung, Yo-Chen Chang, Horng-Jiun Wu, Hung-Chi Lai

**Affiliations:** 1 Department of Ophthalmology, Kaohsiung Medical University Hospital, Kaohsiung Medical University, No. 100, Tzyou 1st Rd., Sanmin Dist., Kaohsiung 80756, Taiwan; sysung52@gmail.com (S.-Y.S.); ycchang@kmu.edu.tw (Y.-C.C.); william.wu.kmuoph@gmail.com (H.-J.W.); 2 Department of Ophthalmology, School of Medicine, Kaohsiung Medical University, Kaohsiung 80708, Taiwan

**Keywords:** polycythemia, proliferative ischemic retinopathy, cigarette smoking, tobacco consumption

## Abstract

Ischemic retinopathy characterized by neovascularization could result from several diseases such as proliferative diabetic retinopathy, hypertensive retinopathy, and retinal vein occlusion. However, ocular ischemic conditions caused by polycythemia have rarely been described. We report the first case of polycythemia-related proliferative ischemic retinopathy in a 41-year-old male heavy smoker who had ocular ischemic condition due to secondary polycythemia. He had sudden loss of vision in his right eye vision with vitreous hemorrhage and a tortuous retinal artery. Tracing back to his history, he was a heavy smoker with more than one pack of cigarettes per day for more than 30 years. Laboratory data revealed elevated levels of hemoglobin (17.7 g/dL) and hematocrit (51.6%) without other abnormal findings. We performed retinal photocoagulation on the neovascular areas and the fibrous membrane. Additionally, the patient was advised to quit smoking. Owing to adherence to this treatment, the patient’s vision gradually recovered. Although rare, polycythemia can cause retinal ischemic events and should be considered as a sight-threatening disease. Photocoagulation is effective on the regression of the neovascular lesion. Most importantly, changes in lifestyle together with smoking cessation are effective in managing secondary polycythemia. In conclusion, prevention and cessation of tobacco consumption helps improve vision health.

## 1. Introduction

Cigarette smoking is the leading cause of preventable diseases, disability, and death worldwide. In the United States, it accounts for more than 480,000 deaths every year, or approximately one in five deaths [1]. Additionally, it causes a substantial economic burden on the individual. During 2010–2014 in the United States, the annual healthcare spending for smoking-related illness was more than US$225 billion based on total personal healthcare expenditures reported in 2014 [2]. Cigarette smoking not only causes systemic vascular diseases, cancers, and lung diseases, which people are more familiar with, but also leads to eye diseases including cataract, age-related macular degeneration, diabetic retinopathy, glaucoma, and Graves’ ophthalmopathy [3]. In such cases, ocular vascular diseases may develop secondary to smoking-related polycythemia.

Polycythemia is a condition characterized by an abnormal increment of the red blood cell mass. In healthy people, the red blood cell mass is in the range of 23–29 mL/kg in females and 26–32 mL/kg in males [4]. Secondary polycythemia is a heterogeneous group of disorders characterized by an elevated red blood cell mass due to either a physiologically appropriate response to tissue hypoxia or physiologically inappropriate secretion of erythropoietin (EPO) and/or other contributing factors. The overproduction of red blood cells can be due to several reasons, ranging from genetic abnormalities to other diseases with secondary causes, including tobacco smoking. In polycythemia, there is an increase in blood viscosity associated with many complications, particularly ischemic events. Ocular manifestations of polycythemia include transient ischemic attacks in the occipital cortex, transient monocular blindness, and vaso-occlusive diseases as reported previously [5].

Proliferative retinopathy is characterized by disease progression by each stage; ischemia of the retinal vessels leads to hypoxia, and as a result of correcting the problem, there is initiation of uncontrolled angiogenesis on the inner surface of the retina or extended to the vitreous body surface, which can subsequently pose a threat to vision owing to retinal traction detachment or vitreous hemorrhage. Several diseases, including proliferative diabetic retinopathy, hypertensive retinopathy, sickle cell retinopathy, and retinal vein occlusion, among others, can cause ischemic retinopathy involving neovascularization. However, polycythemia-related ocular ischemic issues have rarely been described and discussed. In this study, we describe a case of polycythemia-related proliferative ischemic retinopathy, an ocular ischemia condition that was caused by secondary polycythemia. We monitored his ocular condition during treatment, during which his vision recovered with smoking cessation.

## 2. Case Report

A 41-year-old man was referred to our ophthalmology department with a complaint of a sudden decrease in his right eye vision to counting finger 30 cm. One month before the referral, he was admitted to the neurology ward for left-sided thalamic hemorrhage, complicated with poorly controlled hypertension. During admission, he experienced left lower leg pain while walking. Venous thrombosis was noticed in the left popliteal vein, and D-dimer levels were elevated to 4.25 µg/mL. Considering that he had deep vein thrombosis, dabigatran was prescribed as part of prevention and treatment.

At presentation, his visual acuity was counting finger 30 cm in the right eye and 20/20 in the left eye. The intraocular pressures, when measured with a non-contact tonometer, were within the normal limit. Ocular examination findings revealed a normal anterior ocular segment. On fundus examination, the right eye had dense vitreous hemorrhage and the left eye had a normal fundus. However, the patient did not come for further follow-up afterward.

**Figure 1:**
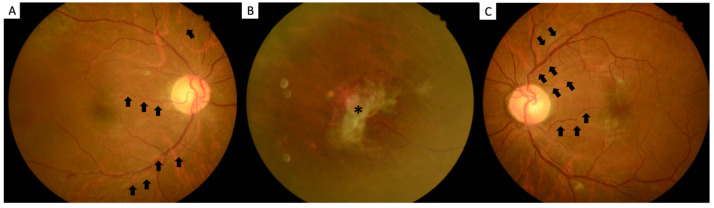
(**A**) Tortuous retinal artery (arrow) and venous sheathing in the right eye; (**B**) Fibrovascular membrane with hemorrhage (asterisk) in the right eye; and (**C**) Tortuous retinal artery (arrow) in the left eye.

**Figure 2:**
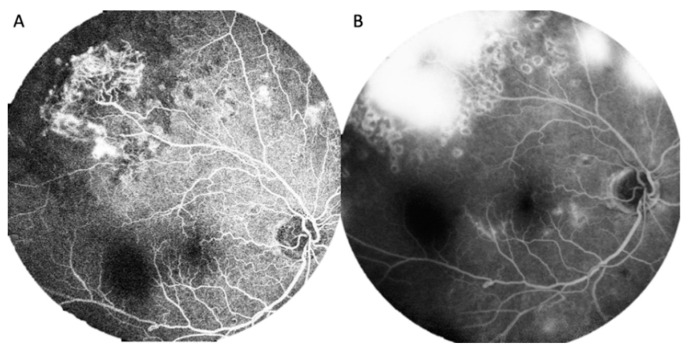
(**A**) Fluorescence stains with surrounding non-perfusion area due to laser treatment at temporal upper and some neovascular lesion on the peripapillary area in the right eye; and (**B**) Diffuse fluorescence leakage at late stage in the right eye.

**Figure 3:**
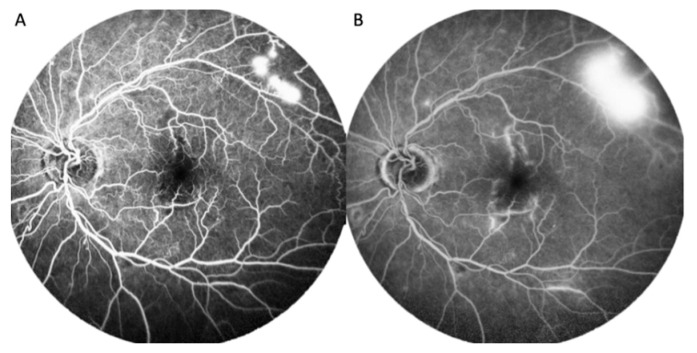
(**A**) Fluorescence stains and leakage at temporal upper; and (**B**) Obvious fluorescence leakage from the temporal upper lesion as well as paramacular neovascular leasions shown at late stage.

More than one year later, he experienced floating blood clots in the right eye and was referred to our clinic for further evaluation. He continued to have a normal ocular anterior segment and normal intraocular pressure. After a decrement of vitreous hemorrhage in the right eye, a tortuous retinal artery, accompanied with venous sheathing and fibro-vascular membrane, was found (Figure 1A,B). Moreover, the tortuous retinal artery was noted in the left eye (Figure 1C). Fundus fluorescence angiography findings revealed a fibro-vascular membrane and some neovascular lesions on the peripapillary area in the right eye (Figure 2A,B). The left eye also had some neovascular lesions (Figure 3A,B), and there was also prolongation of choroidal filling time, delay in retinal arteriovenous transit time, delay in retinal artery phase filling time in both eyes. Macular optical coherent tomography (OCT) (Spectralis OCT, Heidelberg, Germany) findings showed mild macular edema in the right eye and a normal macular structure in the left eye. Owing to a suspected diagnosis of an ocular ischemic condition, his blood sample was assessed, and carotid Doppler and ophthalmic artery Doppler examinations were performed.

Carotid Doppler examination findings showed a normal vessel condition, free of plaques. There was no evident stenosis in the bilateral internal carotid artery and the common carotid artery. Ophthalmic artery Doppler examination showed normal blood flow velocity and flow direction in the bilateral ophthalmic arteries. However, laboratory data revealed normal glycated hemoglobin and elevated levels of hemoglobin (17.7 g/dL) and hematocrit (51.6%). Clinical data at the time of diagnosis and in the subsequent follow-up is listed in Table 1. Under the impression of polycythemia-related neovascular retinopathy, we performed retinal photocoagulation on the neovascular area in both eyes and surrounded the area with a fibrovascular membrane to prevent traction retinal detachment in the right eye. Tracing back his history, he was a heavy smoker and used more than one pack of cigarettes per day for more than 30 years. He also had chronic renal failure. Under a suspected diagnosis of secondary polycythemia, he was advised to quit smoking.

**Figure 4:**
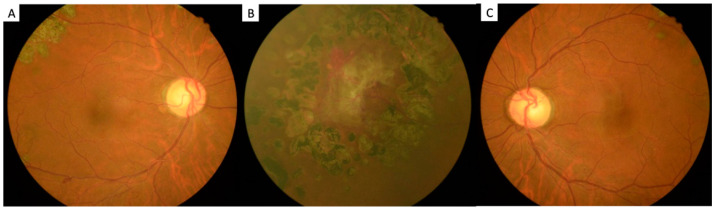
(**A**) Tortuous retinal artery and venous sheathing in the right eye; (**B**) Fibrovascular membrane with hemorrhage surrounded with laser scar in the right eye; and (**C**) Tortuous retinal artery in the left eye.

**Figure 5:**
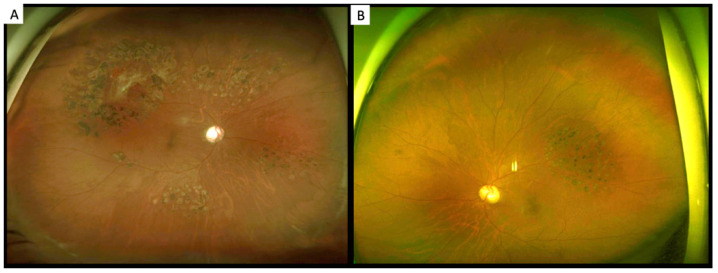
(**A**,**B**) No additional neovascular lesion or fibrovascular membrane formation in both eyes after two years follow up.

After the patient quit smoking, his systemic and ocular condition became stable. He had another laboratory investigation with blood samples, which revealed a decrement in hemoglobin levels (to 16.2 g/dL) and a decrement in hematocrit levels (to 47.6%). Based on the vitreous hemorrhage decrement and macular edema improvement, the patient’s vision was found to gradually recover. After a two-year-follow-up, there were no more systemic and ocular vascular occlusion events (Figure 4 and Figure 5).

## 3. Discussion

Polycythemia is a condition characterized by an increment in red blood cell mass and increased hemoglobin values (16.5 g/dL in males and 16 g/dL in females) and/or hematocrit values (49% in males and 48% in females) in peripheral blood assessment [6]. It is sometimes called erythrocytosis, although erythrocytosis has a different definition of an increased red blood cell count. There are two types of polycythemias. Primary polycythemia occurs as a result of an intrinsic cellular defect. In this type, polycythemia vera is a neoplastic disorder characterized by an increase in the number of erythroid progenitor cells and increased sensitivity to EPO secondary to a Janus kinase mutation. Secondary polycythemia is caused by an elevation of systemic EPO, which is a usual secondary physiologically appropriate response to chronic hypoxia caused by cigarette smoking, hypoxic lung disease, obstructive sleep apnea, cardiopulmonary shunt, and high altitude. Moreover, local renal hypoxia, medications such as testosterone, and pathologic EPO production by malignant disorders can cause secondary polycythemia [4,7]. Increased red blood cell mass resulting from elevated levels of EPO will lead to an increased carrying capacity of the blood to compensate the hypoxic condition. Primary and secondary polycythemia can be differentiated by the level of EPO. A low EPO level (2.9 mU/mL) has a specificity of 92% and sensitivity of 64% for the diagnosis of polycythemia vera; on the contrary, a high EPO level (>15.1 mU/mL) has a specificity of 98% and a sensitivity of 47% for the diagnosis of secondary polycythemia in a cohort study of 125 patients [8].

Cigarette smoking is the major risk factor for hypoxia due to secondary polycythemia. Exposure to more than 7000 chemicals, especially carbon monoxide, nicotine, and tar, is attributed to the development of various diseases in a smoker. Hypoxia is associated with impaired gas exchange and decreased oxygen delivery due to carbon monoxide inhalation and the peripheral vasoconstriction effect of nicotine [9,10,11,12]. As a response to the hypoxic status, there is an increase in the hematological parameters. As reported in several studies, cigarette smoking causes an elevated level of hemoglobin [11,13,14,15], hematocrit [11,15], mean corpuscular volume (MCV) [13,15], and mean corpuscular hemoglobin concentration [13]. It can also cause an elevated level of white blood cells, especially in the sequence of immune responses due to alveolar tissue inflammation and vascular injury [13]. The observational smoking relationships were in the long term for white blood cells and in the short term for red blood cell indices [15]. The hypercoagulable status caused by blood hyperviscosity due to polycythemia, microcirculatory occlusion, and vascular damage predisposed by an increase in leukocytes, endothelium dysfunction related to the inflammatory cascade caused by nicotine, and free radicals increased the risks of vascular disease [13,14,16].

Compared with polycythemia vera, wherein the increased incidence of thromboembolic events has been well established, the association between thromboembolic events and secondary polycythemia is relatively uncertain [17]. In a case-control study of polycythemia vera and polycythemia caused by smoking, 60% of patients with polycythemia vera and 41% of patients with smoking-related polycythemia had at least one thromboembolic problem. Additionally, that study showed that the risk of thromboembolic events with smoking-related polycythemia was not as high as that with polycythemia vera [18]. The mechanism of thrombosis is complicated and is attributed to multiple factors. Exogenous factors are surgery, immobility, pregnancy, and hormone use, whereas endogenous factors are cancer, obesity, and hypercoagulation status, all of which can increase the risk of thrombosis. A case-control study conducted by Nadeem Lynn and colleagues investigated the association between secondary polycythemia and venous thromboembolism and concluded that secondary polycythemia is not an independent risk factor for venous thromboembolism, while obesity in this population of secondary polycythemia may be an associated risk factor partially responsible for the thromboembolic events [19]. This finding indicated the importance of reducing all the consequences caused by cigarette smoking and ceasing other modifiable risk factors rather than treating secondary polycythemia simply by phlebotomy to prevent the patient from acquiring a thromboembolic disease. Timely diagnosis and prompt management are important for preventing further thromboembolic occlusive episodes.

Blood hyper-viscosity associated with polycythemia seems to be the likely cause of the ocular vascular events. In a retrospective study conducted by Yang et al., among 374 patients, the ocular complication rate was 13.6%, with the majority of patients (41.2%) presenting with transient ocular blindness, which is treatable but greatly misdiagnosed. An ocular hemodynamic study with fluorescein angiography aided in identifying delayed choroidal and retinal blood flow in polycythemia patients, accompanied with transient ocular blindness. Moreover, the arm-choroid filling time was found to correlate with hematocrit level and platelet counts as the artery-venous transit time was found to correlate to the hematocrit and hemoglobin levels [20]. With an increased hematocrit level, the narrower width of the plasmatic zone allows for greater platelet–endothelial and platelet–platelet interactions [21]. Venous stasis and hypercoagulable status caused by polycythemia can result in ischemia and hypoxia of the retina as a possible consequence. The retina is one of the most metabolically active tissues. Without adequate circulation, neovascularization, which is clinically characterized by the formation of vessel networks lying on the retinal surface, occurred to compensate for the oxygen and nutrient demands. Ischemic-driven release of cytokines such as vascular endothelial growth factor can promote cell proliferation and migration for neovascularization and increased vascular permeability. With profuse leakiness characteristics, complications such as vitreous hemorrhage, macular edema, neovascular glaucoma, and tractional retinal detachment develop and result in vision loss.

Patients with polycythemia vera reported central retinal artery occlusion [22,23], bilateral anterior ischemic optic neuropathy [21], cotton-wool spot retinopathy [24], and peripheral retinal neovascularization [25] as ocular vascular events. To the best of our knowledge, this is the first case to demonstrate ocular complications caused by secondary polycythemia, and these complications were managed successfully with cessation of smoking. Additionally, the patient was relieved from the systemic hypoxic status and sequencing, which decreased the hemoglobin and hematocrit levels, combined with local treatment for the remaining retinal neovascular lesion, which restored the vision.

Meanwhile, he gained all the benefits of smoking cessation. Cigarette smoking impacts the health systemically and is the leading preventable cause of the disease. It increases the risks of developing cancers, cardiovascular diseases, respiratory diseases, reproductive problems, bony fractures, dental diseases, erectile dysfunction, peptic ulcer diseases, and eye diseases. However, the risks could be reduced by smoking cessation and changes toward a healthy lifestyle. After approximately 12 h, carbon monoxide levels drop to normal levels. For nearly 10 years, the risk of lung cancer falls to half that of a smoker and the risk of cancer in the mouth, throat, esophagus, bladder, cervix, and pancreas decreases. For approximately 15 years, the risk of coronary heart disease remains the same as that of a nonsmoker [3,26]. Smoking cessation at an early age (40 years) has an impressive 90% reduction in the excess risk of death [27]. Studies have shown that smoking cessation will lead to a reversal of abnormal hematological parameters. There were significant reductions in the hemoglobin level, hematocrit level, red blood cell count, and white blood cell count within 24 h after smoking cessation while a decrease in MCV was not found. This indicated that acute changes might be related to the changes in plasma volume associated with decreased catecholamine release induced by nicotine [28]. In another study, all the parameters returned to near-normal values within two years, but some long-term abnormalities lasted for up to five years [29]. Clinically, treatment with phlebotomy, wherein hematocrit is targeted to below 50%, and hydroxycarbamide administration for polycythemia vera patients with transient blindness showed a similar distribution of hematological parameters as that of the control group and resulted in significant improvement of retinal and choroidal circulation for at least two months after treatment [20].

Restoration of vision is not obvious until the development of sight-threatening conditions such as macular edema and vitreous hemorrhage. Not until the visual acuity was worse enough to have an impact on daily life did the long-term effect of the chronic ischemic condition of retina capture the patient’s attention. Moreover, history taking is important in such patients because there may be various possible causes and a definite diagnosis is warranted toward proper management. Our patient experienced two polycythemia-related complications: proliferative ischemic retinopathy and deep vein thrombosis. With the high risk of thrombosis, the patient required systemic anticoagulant treatment. Photocoagulation seemed effective for the regression of neovascular lesions in both eyes of our patient. Changes in lifestyle, such as smoking cessation, are effective for reducing secondary polycythemia and for eliminating the negative impacts on health.

## 4. Conclusions

Patients with polycythemia can present with proliferative ischemic retinopathy, which should be considered as a sight-threatening ocular complication. Early ophthalmic evaluation and intervention may be beneficial to prevent sequential visual loss. Prevention and cessation of tobacco consumption is important for vision health and beneficial for the patient towards the prevention of other systemic diseases.

## Figures and Tables

**Table 1:** Retinopathy aossociated with sickle cell anemia, diabetes mellitus, coagulopathy, thrombophilia disease such as antiphospholipid syndrome and autoimmune disease can be ruled out.

Clinical Data	At Diagnosis	After Recovery
**WBC (/μL)**	8500	10,520
**RBC (×10^6^/μL)**	5.73	5.16
**Platelet (×10^3^/μL)**	201	179
**Hb (g/dL)**	17.7	16.2
**Hct (%)**	51.6	47.6
**MCV (fL)**	90.1	96.3
**RDW (%)**	13.3	13.3
**ESR (mm/h)**	25	15
**HbA (%)**	5.0	5.5
**CHOL(T) (mg/dL)**	239	221
**TG (mg/dL)**	101	
**HDL (mg/dL)**	62.1	51
**LDL (mg/dL)**	141.7	126.7
**PT (second)**	9.7	
**aPTT (second)**	31.3	
**lupus anticoagulant**	negative	
**Anti-cardiolipin antibody**	negative	
**Anti-** **β** **2 glycoprotein-I antibody**	negative	
**ANA**	negative	

## Data Availability

Not applicable.

## References

[1] Centers for Disease Control and Prevention (2022). Current Cigarette Smoking among Adults in the United States.

[2] Xu X., Shrestha S.S., Trivers K.F., Neff L., Armour B.S., King B.A. (2021). U.S. healthcare spending attributable to cigarette smoking in 2014. Prev. Med..

[3] Office of the Surgeon General, Office on Smoking and Health (2004). Reports of the Surgeon General. The Health Consequences of Smoking: A Report of the Surgeon General.

[4] Hocking W.G., Golde D.W. (1989). Polycythemia: Evaluation and management. Blood Rev..

[5] Blood A.M., Lowenthal E.A., Nowakowski R.W. (1997). Retinopathy secondary to anemia from myeloid metaplasia in polycythemia vera. J. Am. Optom. Assoc..

[6] Tefferi A., Barbui T. (2020). Polycythemia vera and essential thrombocythemia: 2021 update on diagnosis, risk-stratification and management. Am. J. Hematol..

[7] Mithoowani S., Laureano M., Crowther M.A., Hillis C.M. (2020). Investigation and management of erythrocytosis. CMAJ.

[8] Mossuz P., Girodon F., Donnard M., Latger-Cannard V., Dobo I., Boiret N., Lecron J.C., Binquet C., Barro C., Hermouet S. (2004). Diagnostic value of serum erythropoietin level in patients with absolute erythrocytosis. Haematologica.

[9] Fricker M., Goggins B.J., Mateer S., Jones B., Kim R.Y., Gellatly S.L., Jarnicki A.G., Powell N., Oliver B.G., Radford-Smith G. (2018). Chronic cigarette smoke exposure induces systemic hypoxia that drives intestinal dysfunction. JCI Insight.

[10] Jensen J.A., Goodson W.H., Hopf H.W., Hunt T.K. (1991). Cigarette smoking decreases tissue oxygen. Arch. Surg..

[11] Alkhedaide A.Q. (2020). Tobacco smoking causes secondary polycythemia and a mild leukocytosis among heavy smokers in Taif City in Saudi Arabia. Saudi J. Biol. Sci..

[12] Moodley T., Mannaru K.T., Hugo A., Lines J.A., Van der Merwe J.M., Ramparsad N., Holland N.S. (2021). Secondary polycythaemia with elevated carbon monoxide levels due to hookah pipe smoking: A public health concern. S. Afr. Med. J..

[13] Malenica M., Prnjavorac B., Bego T., Dujic T., Semiz S., Skrbo S., Gusic A., Hadzic A., Causevic A. (2017). Effect of Cigarette Smoking on Haematological Parameters in Healthy Population. Med. Arch..

[14] AlQahtany F.S., Algahtani F.H., Alshebly M.M., Madkhaly F.M., Ghandour M.K., Almalki J.H., AlOtaibi W.S., Salim A., Mendoza F.C. (2020). Association between cigarette & shisha smoking and the severity of polycythemia: A cross sectional study. Saudi J. Biol. Sci..

[15] Pedersen K.M., Çolak Y., Ellervik C., Hasselbalch H.C., Bojesen S.E., Nordestgaard B.G. (2019). Smoking and Increased White and Red Blood Cells. Arterioscler. Thromb. Vasc. Biol..

[16] Kondo T., Nakano Y., Adachi S., Murohara T. (2019). Effects of Tobacco Smoking on Cardiovascular Disease. Circ. J..

[17] Bhatt V.R. (2014). Secondary polycythemia and the risk of venous thromboembolism. J. Clin. Med. Res..

[18] Schwarcz T.H., Hogan L.A., Endean E.D., Roitman I.T., Kazmers A., Hyde G.L. (1993). Thromboembolic complications of polycythemia: Polycythemia vera versus smokers’ polycythemia. J. Vasc. Surg..

[19] Nadeem O., Gui J., Ornstein D.L. (2013). Prevalence of venous thromboembolism in patients with secondary polycythemia. Clin. Appl. Thromb. Hemost..

[20] Yang H.S., Joe S.G., Kim J.G., Park S.H., Ko H.S. (2013). Delayed choroidal and retinal blood flow in polycythaemia vera patients with transient ocular blindness: A preliminary study with fluorescein angiography. Br. J. Haematol..

[21] Tönz M.S., Rigamonti V., Iliev M.E. (2008). Simultaneous, bilateral anterior ischemic optic neuropathy (AION) in polycythemia vera: A case report. Klin. Mon. Augenheilkd..

[22] Rao K., Shenoy S.B., Kamath Y., Kapoor S. (2016). Central retinal artery occlusion as a presenting manifestation of polycythaemia vera. BMJ Case Rep..

[23] Ganesan S., Raman R., Sharma T. (2017). Polycythemia causing posterior segment vascular occlusions. Oman J. Ophthalmol..

[24] Ahn B.Y., Choi K.D., Choi Y.J., Jea S.Y., Lee J.E. (2007). Isolated monocular visual loss as an initial manifestation of polycythemia vera. J. Neurol. Sci..

[25] Krishnan R. (2009). Peripheral retinal neovascularization associated with polycythemia rubra vera. Jpn. J. Ophthalmol..

[26] World Health Organization (2020). Tobacco: Health Benefits of Smoking Cessation Q&A.

[27] Gallucci G., Tartarone A., Lerose R., Lalinga A.V., Capobianco A.M. (2020). Cardiovascular risk of smoking and benefits of smoking cessation. J. Thorac. Dis..

[28] Bain B.J., Rothwell M., Feher M.D., Robinson R., Brown J., Sever P.S. (1992). Acute changes in haematological parameters on cessation of smoking. J. R. Soc. Med..

[29] Van Tiel E., Peeters P.H., Smit H.A., Nagelkerke N.J., Van Loon A.J., Grobbee D.E., Bueno-de-Mesquita H.B. (2002). Quitting smoking may restore hematological characteristics within five years. Ann. Epidemiol..

